# Prediction of Structural Behavior of Continuous Reinforced Concrete Beams with Hybrid CFRP-Steel Bars

**DOI:** 10.3390/ma15217542

**Published:** 2022-10-27

**Authors:** Miao Pang, Yi Dong, Xing Liu, Wei Sun, Tiejiong Lou

**Affiliations:** 1 Department of Civil Engineering, Zhejiang University, Hangzhou 310058, China; 2 School of Civil Engineering and Architecture, Wuhan University of Technology, Wuhan 430070, China; 3 College of Civil Engineering, Huaqiao University, Xiamen 361021, China; 4 Centre for Mechanical Engineering, Materials and Processes (CEMMPRE), University of Coimbra, 3030-788 Coimbra, Portugal

**Keywords:** CFRP bars, hybrid reinforcement, continuous beams, flexure

## Abstract

The present investigation aims to identify the flexural performance of two-span concrete beams reinforced with hybrid carbon fiber reinforced polymer (CFRP) and steel bars. By applying a finite element analysis, a comprehensive numerical assessment is performed. The investigated variables are *A_f_*/*A_r_* (*A_f_* = area of CFRP bars; *A_r_* = total area of CFRP/steel bars), load pattern (symmetrical and unsymmetrical loading) and load type (center-point, third-point and uniform loading). The results show that beams with *A_f_*/*A_r_* of 0.25 show 16.0% and 11.3% higher ultimate load at symmetrical and unsymmetrical loading, respectively, than beams with *A_f_*/*A_r_* of 0.0 (i.e., beams with steel bars), but the change in ultimate load is not apparent when varying *A_f_*/*A_r_* between 0.25 and 1.0. Unsymmetrical loading causes 6.0–15.0% greater deflection capacities than the symmetrical one. When *A_f_*/*A_r_* increases from 0.0 to 1.0, moment redistribution at symmetrical loading is decreased significantly by 62%, while the redistribution variation is marginal at unsymmetrical loading. In addition, the applicability of two equations based on the ultimate strain in tensile bars for predicting moment redistribution is evaluated. It is generally shown that these equations can account for the influence of *A_f_*/*A_r_* and load type.

## 1. Introduction

Reinforced concrete (RC) is widespread in civil infrastructures around the world, accompanying the progressive development of concrete materials [1,2,3]. Conventional steel bars in RC elements are subject to corrosive damage, resulting in progressive deterioration of the structure [4]. Fiber reinforced polymer (FRP) has the advantages of non-corrosiveness and high strength, and is recognized as an alternative to steel bars [5]. These composite materials are widely employed for reinforcing or strengthening concrete structures [6,7,8]. However, FRP is linearly elastic until rupture without yielding, thereby raising concerns about the flexural ductility of RC elements with FRP bars [9,10]. In continuous RC beams, the use of FRP bars may also negatively influence the ability to redistribute moments as the redistribution is closely related to flexural ductility [11,12,13,14]. Moreover, FRP commonly has a low elastic modulus, which may lead to excessive deflections [15,16,17] and crack width [18,19] of RC beams with FRP bars at the serviceability limit state. Experimental research shows that the crack pattern of these beams is positively influenced by the local confinement along the anchorage length of FRP bars [20].

Hybrid FRP-steel bar systems have recently been introduced to take advantage of the merits of both steel and FRP materials. In this system, FRP bars are usually located at the outer level and steel bars at the inner level to achieve adequate protection against corrosion. Extensive experimental, numerical and theoretical works on single-span RC beams with hybrid FRP-steel bars have been conducted [21,22,23,24,25,26]. Previous works have generally shown that RC beams with hybrid bars exhibit improved ductility and deflection control, compared to RC beams with FRP bars. Meanwhile, RC beams with hybrid bars show better durability and higher ultimate load than RC beams with steel bars. Recent studies by Gemi et al. [27,28] demonstrated that specimens with hybrid bars exhibited superior behavior including in their energy-dissipation and load-carrying capacities, and, therefore, the use of hybrid bars in RC elements is strongly recommended.

In practice, continuous beams are more common than simply supported ones. However, research on continuous RC beams with hybrid bars is scarce. Araba and Ashour [29] conducted an experimental study to investigate the flexural response of RC continuous beams with hybrid GFRP-steel bars. Their tests contained five two-span RC specimens with hybrid bars, one control RC specimen with steel bars and one control RC specimen with GFRP bars. The tested parameters included the number of longitudinal bars and the ratio of steel to GFRP bar area. Their tests showed that adding GFRP bars led to a higher ultimate load but a lower ductility, and that adding steel bars led to an improved ductile behavior but a decrease in load increment after steel yielding. Limited redistribution of moments was observed in their test specimens. In a later study, Almahmood et al. [30] tested six continuous T-beams, including five RC beams with hybrid GFRP-steel bars and one RC control beam with GFRP bars. They concluded that RC beams with hybrid bars showed better flexural performance in terms of flexural stiffness, ductility, crack width and deflection control, compared to the RC beam with GFRP bars. However, adding steel bars to RC beams with GFRP bars did not obviously enhance the ability to redistribute moments. Akiel et al. [31] tested 12 two-span specimens, of which six specimens were reinforced with BFRP-steel hybrid bars. Their test results showed that RC specimens with hybrid bars exhibited comparable deformation but low moment redistribution when compared to RC specimens with BFRP bars.

The above literature review shows that few works on RC continuous beams with hybrid bars are available. Further investigation on the topic is therefore necessary. This paper aims to improve the understanding of the flexural behavior of continuous RC beams with hybrid CFRP-steel bars. A numerical study is performed on two-span RC beams. The main investigated variables are the hybrid level (ranging from 0.0 to 1.0), the load pattern (symmetrical and unsymmetrical loading) and the load type (center-point, third-point and uniform loading). Comprehensive aspects of behavior are assessed and relevant findings are concluded.

## 2. Numerical Method

**Figure 1:**
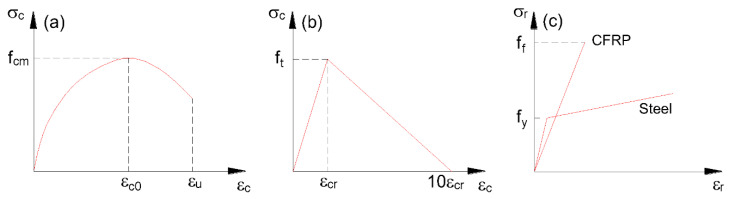
Stress–strain curves of materials. (**a**) Concrete in compression; (**b**) concrete in tension; (**c**) reinforcement bars.

The present numerical study on continuous RC beams with hybrid bars is performed by applying a finite element analysis (FEA) integrated with nonlinear material laws. As illustrated in Figure 1a, the stress–strain relationship for concrete in compression is expressed as follows [32]:

(1) $$\frac{\sigma_{c}}{f_{cm}}=\frac{k(\varepsilon_{c}/\varepsilon_{c0})-{\left(\varepsilon_{c}/\varepsilon_{c0}\right)}^{2}}{1+(k-2)(\varepsilon_{c}/\varepsilon_{c0})}.$$

where *σ_c_* and *ε_c_* are concrete stress and strain, respectively; *f_cm_* = *f_ck_* + 8, in which *f_cm_* and *f_ck_* are concrete mean and characteristic cylinder compressive strengths, respectively; *ε_c_*_0_ is the strain at peak stress; *k* is a coefficient. The above equation is valid when *ε_c_* ≤ *ε_u_*, in which *ε_u_* is the ultimate concrete compressive strain. The concrete in tension shows elastic behavior before cracking and linearly tension-stiffening behavior after cracking, as illustrated in Figure 1b, where *f_t_* is the concrete tensile strength; and *ε_cr_* is the cracking strain. The stress–strain (*σ_r_* − *ε_r_*) curves for reinforcement bars are shown in Figure 1c. CFRP bars are linear-elastic until their rupture strength *f_f_*; and steel bars are linear-elastic until their yield strength *f_y_*, followed by linear strain-hardening with a modulus of 0.015 times the elastic modulus.

The beam is divided into two-node Timoshenko beam elements. Each node has two degrees of freedom, namely transverse displacement *w* and rotation *θ*. The displacement/rotation within the element is obtained using linear interpolation. By applying the principle of virtual work, the following element equilibrium equations are established:

(2) $$P^{e}=(K_{b}^{e}+K_{s}^{e})u^{e}$$

(3) $$K_{b}^{e}=\int_{l}B_{b}^{T}(EI)B_{b}dx,\;K_{s}^{e}=\int_{l}B_{s}^{T}(GA/k_{s})B_{s}dx$$

(4) $$B_{b}=\left[\begin{array}{cccc} 0 & \frac{1}{l} & 0 & -\frac{1}{l} \end{array}\right],\;B_{s}=\left[\begin{array}{cccc} -\frac{1}{l} & -1+\frac{x}{l} & \frac{1}{l} & -\frac{x}{l} \end{array}\right]$$

where ***P****^e^* is the element equivalent nodal loads; ***u****^e^* is the element nodal displacements; (*EI*) and (*GA*) are flexural stiffness and shear stiffness, respectively; *k_s_* is the shear correction factor; and *l* is the element length.

The equilibrium equations for the structure are solved by the incremental-iterative method. During the solution process, when concrete or reinforcement reaches the ultimate capacity, the analysis is terminated. A complete analysis of continuous beams involves two load steps. In the first step, only self-weight of the beams is applied, and a load-control incremental method is employed for the numerical solution. In the second step, external loads are applied until the failure of the beams, and a displacement-control incremental method is employed for the numerical solution. Details of the numerical procedure are reported in [33]. A computer program was developed and implemented in Fortran. The proposed numerical method was verified by comparison with experimental data of a large number of continuous RC beams with steel and/or CFRP bars [11,12,33,34].

## 3. Numerical Investigation

**Figure 2:**
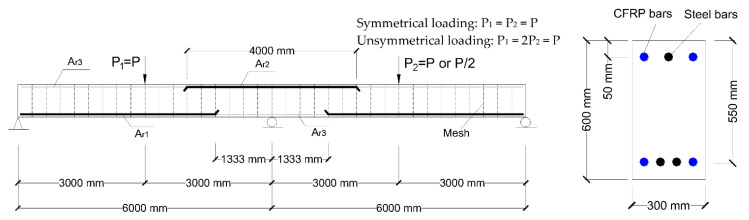
RC continuous beams with hybrid CFRP-steel bars and the finite element mesh.

RC beams with two identical spans symmetrically or unsymmetrically subjected to center-point loading, as shown in Figure 2, are used in this part of the investigation. For symmetrical loading, *P*_1_ = *P*_2_ = *P*, where *P* is the applied load; *P*_1_ and *P*_2_ are the applied load at the left-hand and right-hand spans, respectively. For unsymmetrical loading, *P*_1_ = 2*P*_2_ = *P*. The areas of tensile bars at midspan and center support, *A_r_*_1_ and *A_r_*_2_, are 3000 and 2000 mm^2^, respectively. The area of compressive bars, *A_r_*_3_, is 600 mm^2^. Different levels of hybrid bars (*A_f_*/*A_r_*) are used, i.e., *A_f_*/*A_r_* = 0.0, 0.25, 0.5, 0.75 and 1.0, where *A_f_* is the area of CFRP bars, and *A_r_* is the total area of CFRP/steel bars. The beams are reinforced only with steel bars in the case for *A_f_*/*A_r_* = 0.0, and CFRP bars in the case for *A_f_*/*A_r_* = 1.0. The rupture strength, rupture strain and elastic modulus of CFRP bars are 1450 MPa, 1.09% and 133 GPa, respectively; the yield strength and elastic modulus of steel bars are 530 MPa and 200 GPa, respectively. The cylinder compressive strength of concrete (*f_ck_*) is 30 MPa. Prior to the numerical parametric investigation, a mesh sensitivity analysis is carried out. The analysis shows that a smaller size of beam elements causes a smaller plastic rotation, but it also leads to a larger element curvature and hence a smaller element stiffness. A larger curvature would also counteract the influence of element size on the plastic rotation (the integral of curvatures on the length of the yielded element). In other words, the mesh size influences both the plastic rotation and flexural stiffness of the critical elements. As a result, moment redistribution is also size-dependent due to the combined effects of plastic rotation and flexural stiffness. In the current finite element mesh, the two-span beam is equally divided into 36 elements having a length of 333.33 mm each, as illustrated in Figure 2.

### 3.1. Failure and Cracking Modes

**Figure 3:**
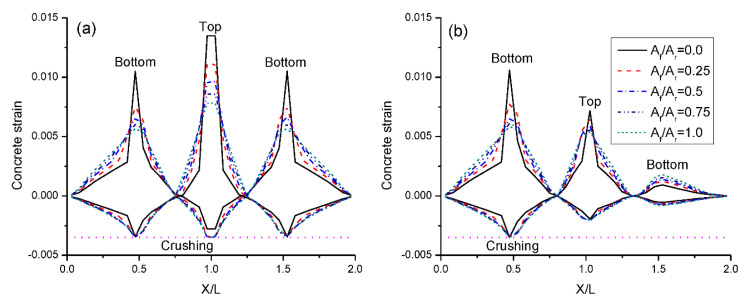
Concrete strain distribution: (**a**) symmetrical loading; (**b**) unsymmetrical loading. (*X*/*L* = ratio of distance from end support to span).

Figure 3 shows the concrete strain distribution at ultimate under symmetrical/unsymmetrical loading. Crushing failure takes place once concrete gets to its ultimate compressive strain. At symmetrical loading, the RC beam with steel bars (i.e., *A_f_*/*A_r_* = 0.0) is crushed at midspan while the concrete strain at center support is still well below its ultimate capacity. The RC beams with CFRP and hybrid CFRP-steel bars (i.e., *A_f_*/*A_r_* > 0.0) are crushed at center support and the concrete strain at midspan is close to its ultimate capacity. In addition, these beams exhibit obviously larger compressive strain over non-critical regions than the RC beam with steel bars as shown in Figure 3a, indicating better exploitation of these regions. At unsymmetrical loading, all the beams fail by concrete crushing at the critical midspan, while at failure concrete at center support is far below its ultimate capacity. The RC beam with steel bars exhibits lower exploitation of the non-critical regions than beams reinforced with CFRP and hybrid bars, as shown in Figure 3b.

At failure, the crack mode can be represented by tensile strain distribution (see Figure 3). The RC beams with steel bars exhibit irregular strain distribution, i.e., large tensile strains in the critical sections against small ones in other zones, suggesting crack concentration in the beams. By using hybrid or CFRP bars, the crack concentration is effectively mitigated. It is shown that an increase in the *A_f_*/*A_r_* ratio results in a marked reduction in the crack width at the critical sections.

### 3.2. Moment-Curvature Behavior

**Figure 4:**
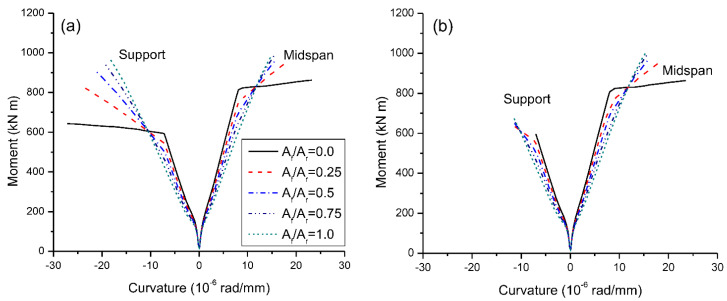
Moment-curvature behavior: (**a**) symmetrical loading; (**b**) unsymmetrical loading.

Figure 4 shows the moment-curvature behavior under symmetrical/unsymmetrical loading. As expected, the moment-curvature behavior of RC beams with steel bars, except at center support under unsymmetrical loading, consists of three distinct stages. The first stage is characterized by an elastic behavior up to cracking, followed by the second stage featured by a linear behavior with substantially reduced stiffness up to yielding. In the third stage, the flexural stiffness is dramatically decreased and there is a linear moment-curvature behavior up to the ultimate. At unsymmetrical loading, steel at center support has not yielded at ultimate. In contrast, the moment-curvature behavior of RC beams with CFRP bars experiences two distinct stages, i.e., elastic stage and elastically cracked stage. The RC beams with hybrid bars exhibit nonlinear moment-curvature behavior after cracking, while the level of nonlinearity is dependent on the hybrid level, i.e., the higher the hybrid level, the less apparent the nonlinearity. It is noted that a higher *A_f_*/*A_r_* ratio leads to a lower yielding moment, attributed to that in this stage the contribution of CFRP bars is lower than that of steel bars.

**Figure 5:**
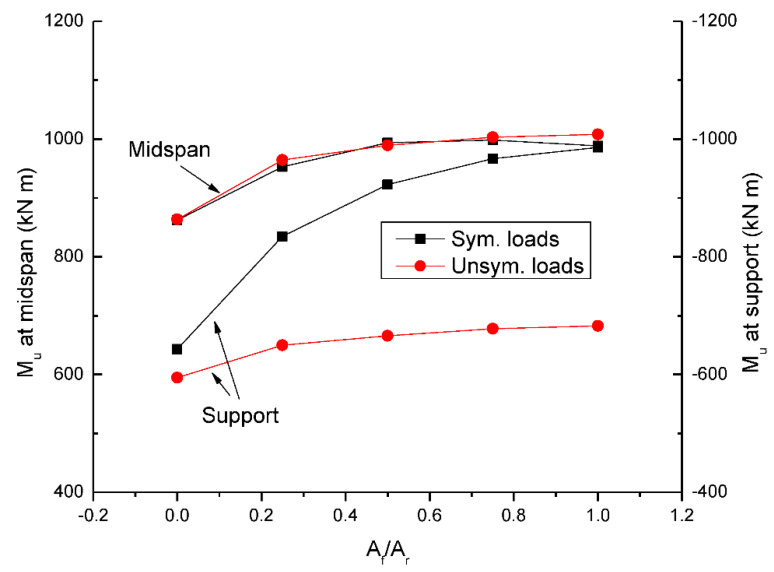
Change in ultimate moment (*M_u_*) with the hybrid level.

Figure 5 shows the change in ultimate moment with varying *A_f_*/*A_r_*. At symmetrical loading, the support moment in the RC beam with steel bars (i.e., *A_f_*/*A_r_* = 0.0) is significantly lower than the midspan moment, attributed to the fact that the tensile bar area at center support is only 66.7% of that at midspan. As *A_f_*/*A_r_* increases, the ultimate moment tends to increase while the increase in support moment is substantially quicker than that in midspan moment. This can be explained by the fact that CFRP bars develop higher stress than steel bars, especially notable at center support. Consequently, the difference between midspan and support moments is reduced as *A_f_*/*A_r_* increases. In the RC beam with CFRP bars, (i.e., *A_f_*/*A_r_* = 1.0), the support moment appears to be comparable to the midspan moment. At unsymmetrical loading, the support moment is around 67% of the midspan moment, regardless of the *A_f_*/*A_r_* ratio. The midspan moment by unsymmetrical loading is comparable to that of symmetrical loading. However, unsymmetrical loading leads to a lower support moment than symmetrical loading, and the moment difference is increasingly notable with increasing *A_f_*/*A_r_*.

**Figure 6:**
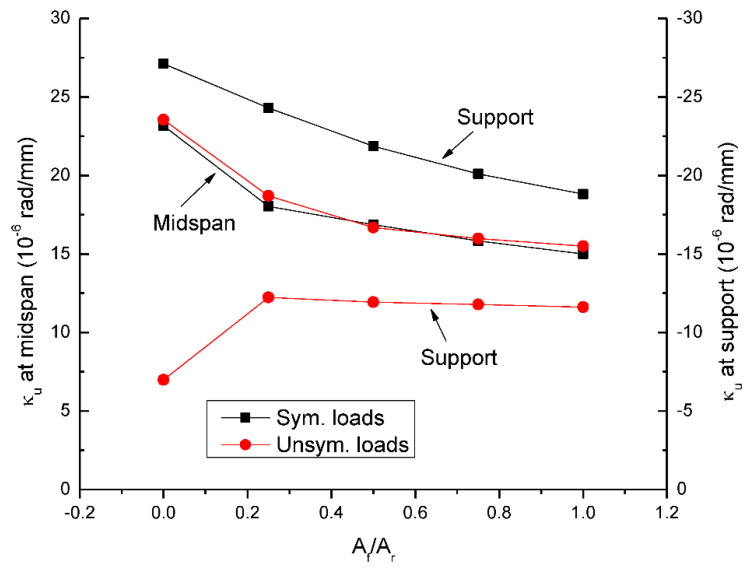
Change in ultimate curvature (*κ_u_*) with the hybrid level.

Figure 6 shows the change in ultimate curvature with varying *A_f_*/*A_r_*. It is seen that the curvature in critical sections decreases as *A_f_*/*A_r_* increases. In addition, the midspan curvature is lower (higher) than the support curvature at symmetrical (unsymmetrical) loading. At a given *A_f_*/*A_r_* ratio, unsymmetrical loading leads to comparable midspan curvature but remarkably lower support curvature, when compared to symmetrical loading.

### 3.3. Load-Deflection Behavior

**Figure 7:**
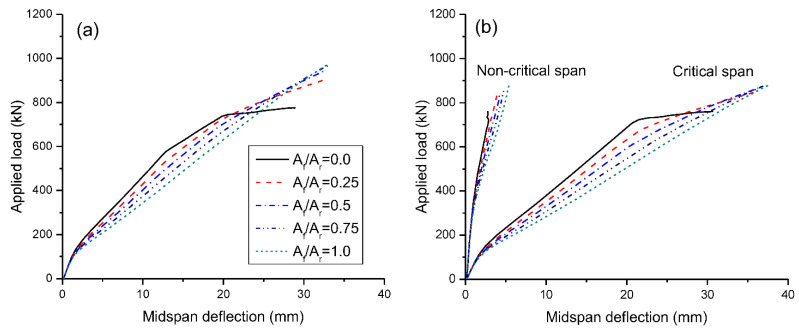
Load-deflection behavior: (**a**) symmetrical loading; (**b**) unsymmetrical loading.

Figure 7 shows the load-deflection behavior under symmetrical/unsymmetrical loading. Prior to failure, the load-deflection behavior of RC beams with CFRP bars (i.e., *A_f_*/*A_r_* = 1.0) is primarily affected by concrete cracking, and also by steel yielding for RC beams with steel and hybrid bars (i.e., *A_f_*/*A_r_* < 1.0). However, the yielding in RC beams with hybrid bars is decreasingly apparent as *A_f_*/*A_r_* increases. It is also observed that beams under symmetrical loading show stiffer behavior than beams under unsymmetrical loading. At unsymmetrical loading, the deflection of partially loaded span is marginal.

**Figure 8:**
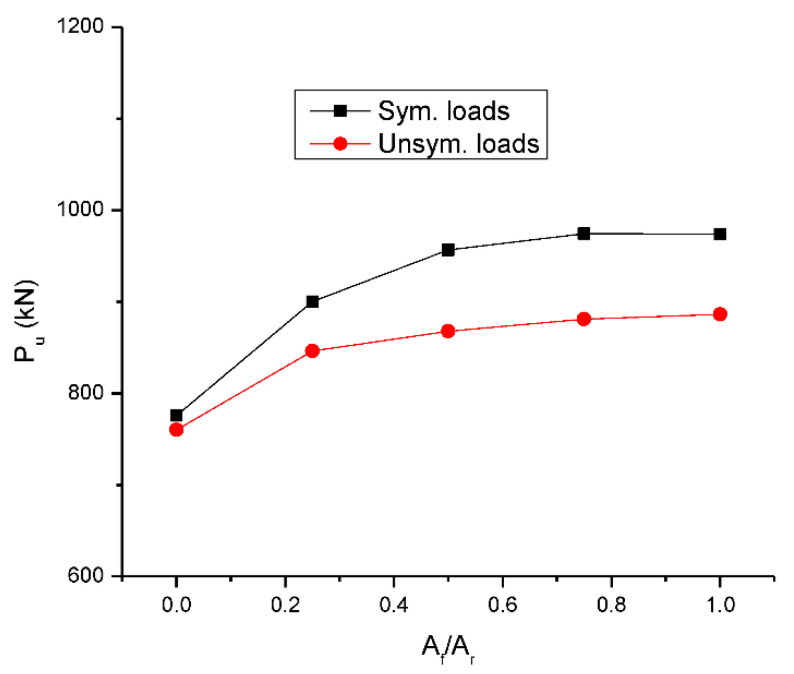
Change in ultimate load (*P_u_*) with the hybrid level.

Figure 8 shows the change in ultimate load with varying *A_f_*/*A_r_*. It is seen that the ultimate load tends to increase with increasing *A_f_*/*A_r_*, attributed to higher stress developed in CFRP bars than that in steel bars. The increase in ultimate load is more apparent for symmetrical loading than for unsymmetrical loading. Increasing *A_f_*/*A_r_* from 0.0 to 0.25 results in an obvious increase in ultimate load by 16.0% for symmetrical loading and 11.3% for unsymmetrical loading. With continuous increase in *A_f_*/*A_r_*, the enhancement in ultimate load is rather limited.

**Figure 9:**
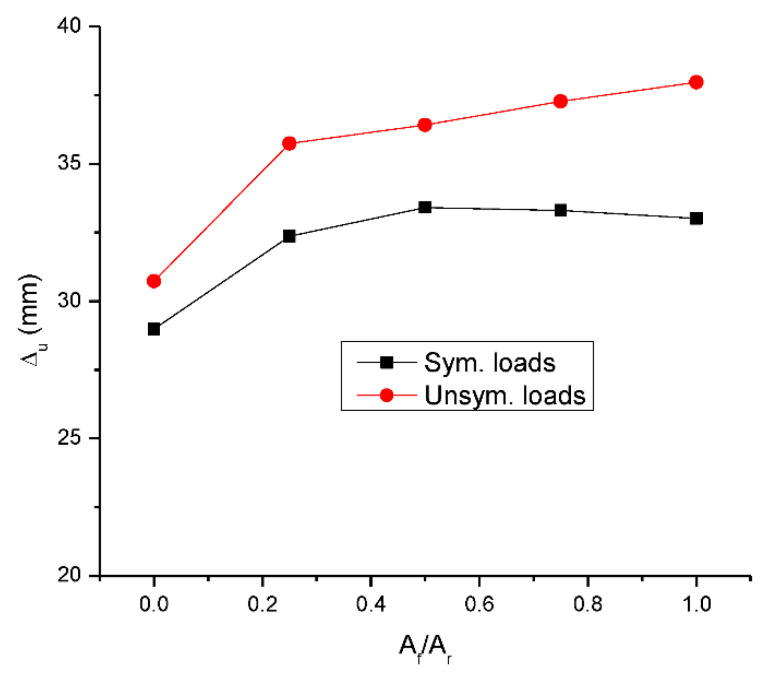
Change in ultimate deflection (Δ*_u_*) with the hybrid level.

Figure 9 shows the change in ultimate deflection with varying *A_f_*/*A_r_*. The ultimate deflection is increased by 11.7% at symmetrical loading and by 16.3% at unsymmetrical loading when *A_f_*/*A_r_* increases from 0.0 to 0.25. With continuous increases in *A_f_*/*A_r_*, the ultimate deflection hardly changes (at symmetrical loading) or slightly increases (at unsymmetrical loading). At a given *A_f_*/*A_r_* ratio, unsymmetrical loading leads to 6.0–15.0% higher ultimate deflection than symmetrical loading.

### 3.4. Strain in Tensile Bars

**Figure 10:**
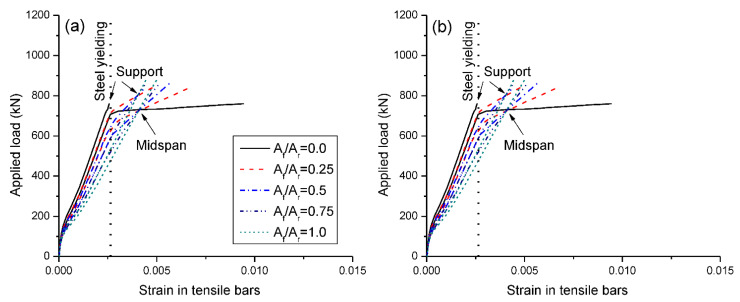
Load versus bar strain: (**a**) symmetrical loading; (**b**) unsymmetrical loading.

Figure 10a,b show the load versus tensile bar strain behavior of beams under symmetrical and unsymmetrical loading, respectively. The beams with different *A_f_*/*A_r_* ratios exhibit the same strain development behavior until cracking. After cracking, a higher *A_f_*/*A_r_* ratio leads to a quicker increase in bar strain. In the case for *A_f_*/*A_r_* = 1.0 (i.e., RC beam with CFRP bars), the development of post-cracking strain in bars shows a linear manner until the ultimate. For beams fully or partially reinforced with steel bars (*A_f_*/*A_r_* < 1.0), the strain development in bars is influenced by the steel yielding, while the degree of influence diminishes as *A_f_*/*A_r_* increases, as demonstrated in Figure 10. At ultimate, all steel bars have yielded except at center support of the RC beam with steel bars under unsymmetrical loading, in which steel bars have not reached (but are close to) their yield strain.

**Figure 11:**
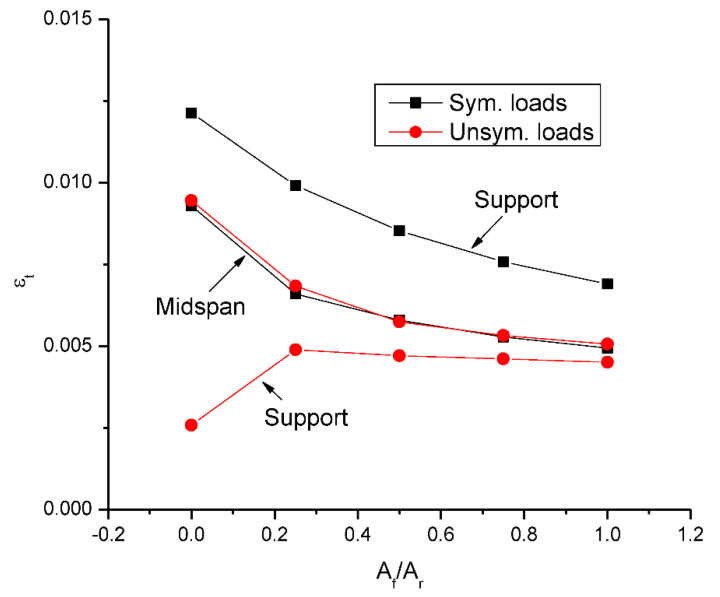
Change in ultimate strain in tensile bars (*ε_t_*) with the hybrid level.

Figure 11 shows the change in ultimate strain in tensile bars with varying *A_f_*/*A_r_*. At symmetrical loading, the ultimate bar strains at midspan and center support decrease as *A_f_*/*A_r_* increases. At a given *A_f_*/*A_r_* ratio, the bar strain at midspan is substantially (around 30%) lower than that at midspan. At unsymmetrical loading, the bar strain at midspan tends to decrease as *A_f_*/*A_r_* increases, while the bar strain at center support is remarkably increased by 89.4% to 4883 με when increasing *A_f_*/*A_r_* from 0.0 to 0.25, and tends to stabilize thereafter. Unsymmetrical loading leads to comparable bar strain at midspan but substantially lower bar strain at center support compared to symmetrical loading.

### 3.5. Development of Bending Moments

**Figure 12:**
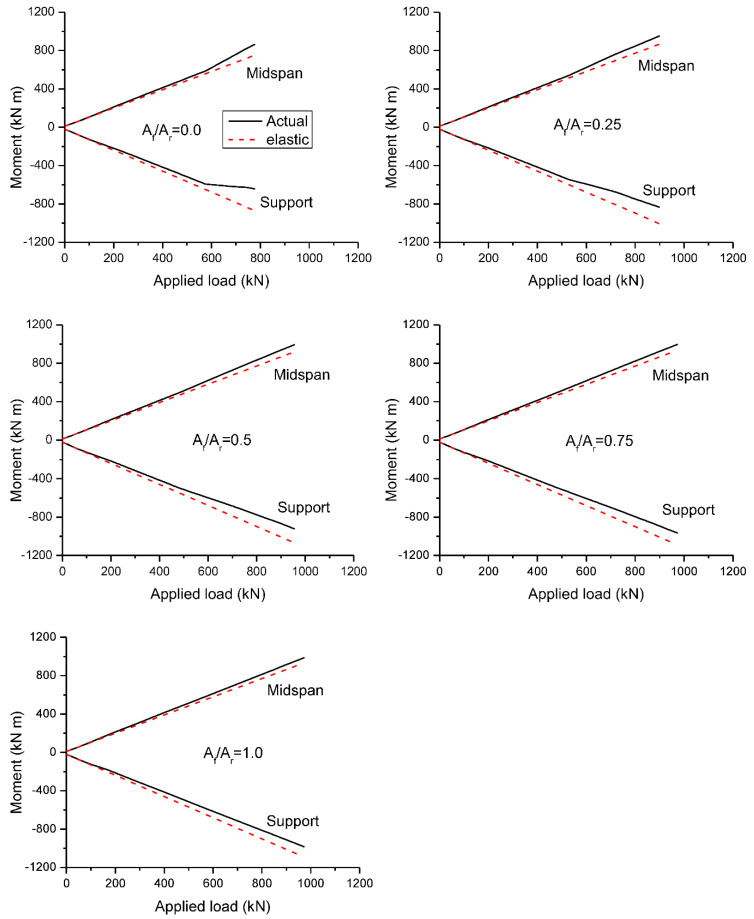
Load-moment behavior at symmetrical loading.

**Figure 13:**
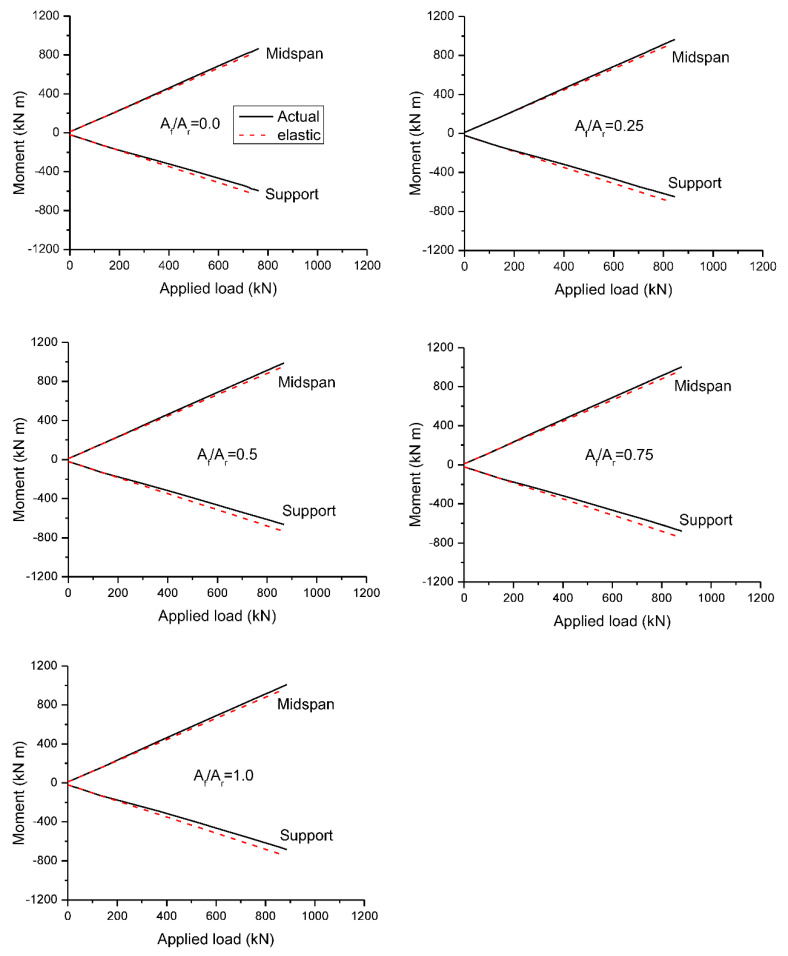
Load-moment behavior at unsymmetrical loading.

The load-moment behavior under symmetrical loading is shown in Figure 12, and the behavior under unsymmetrical loading is illustrated in Figure 13. The results generated from both nonlinear and elastic analyses are presented. There is a linear relationship between the elastic moment and the applied load. Prior to cracking, the moment development generated by the nonlinear analysis is exactly the same as that of the elastic analysis. After cracking, the moment development no longer remains linear because of the occurrence of redistribution.

At symmetrical loading, as cracks occur firstly at center support, bending moments are redistributed away from this critical section. Consequently, the moment growth is slower at center support and quicker at midspan. The difference between actual and elastic moments at center support appears to be more apparent than that at midspan, as illustrated in Figure 12. The beams with various *A_f_*/*A_r_* ratios exhibit approximately identical load-moment behavior until yielding of steel bars (if any) at center support. After that, the moment development is strongly dependent on the *A_f_*/*A_r_* ratio, i.e., the higher the *A_f_*/*A_r_* ratio, the smaller the deviation between actual and elastic moments.

Figure 13 shows that at unsymmetrical loading, the deviation between actual and elastic moments appears to be insignificant during the entire inelastic range of loading, indicating minimal moment redistribution. This is attributed to the combined effect of load pattern and reinforcement arrangement. On the one hand, moments tend to be redistributed from the critical midspan to the center support in the case of unsymmetrical loading while, on the other hand, moments tend to be redistributed from the lower-reinforced section (center support) to the higher-reinforced section (midspan).

### 3.6. Moment Redistribution

**Figure 14:**
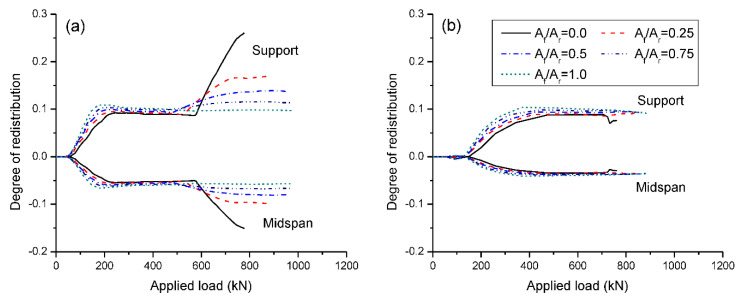
Load versus moment redistribution: (**a**) symmetrical loading; (**b**) unsymmetrical loading.

Figure 14 shows the load versus moment redistribution for beams under symmetrical/unsymmetrical loading. Moment redistribution is quantified by: $\beta =1-(M/M_{e})$, where *β* is the degree of moment redistribution; *M* is the moment obtained by nonlinear analysis; and *M_e_* is the elastic moment. In the case of symmetrical loading, the RC beam with CFRP bars (i.e., *A_f_*/*A_r_* = 1.0) shows three-stage redistribution behavior as illustrated in Figure 14a. The first stage corresponds to the elastic behavior without any redistribution of moments. The second stage, starting from cracking at center support, is characterized by a fast development in moment redistribution. The third stage, initiated by stabilizing of crack development, features stabilizing of moment redistribution. For beams fully or partially reinforced with steel bars (i.e., *A_f_*/*A_r_* < 1.0), there are two additional stages triggered by first and second steel yielding, respectively. Moment redistribution resumes an increase on first steel yielding. This increase is remarkable for the RC beam with steel bars (i.e., *A_f_*/*A_r_* = 0.0) while it becomes less apparent for RC beams with hybrid bars. After second yielding, moment redistribution remains almost unchanged. In the case of unsymmetrical loading, the influence of steel yielding on the development of moment redistribution appears to be negligible. Consequently, the beams with different *A_f_*/*A_r_* ratios exhibit very similar redistribution behavior as shown in Figure 14b.

**Figure 15:**
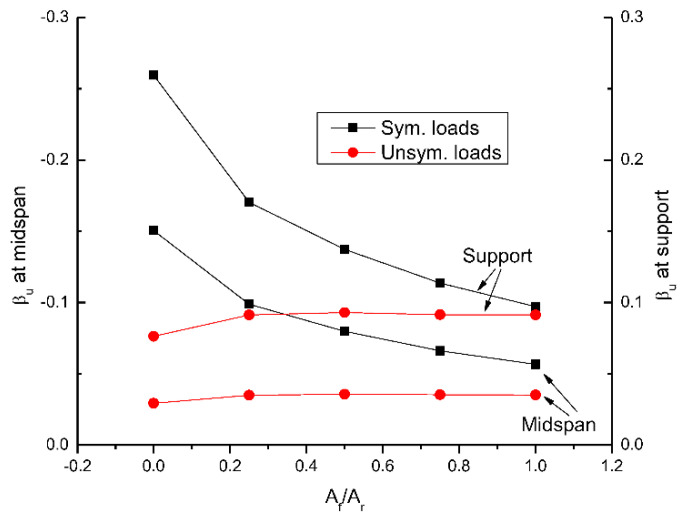
Change in moment redistribution at ultimate (*β_u_*) with the hybrid level.

Figure 15 shows the change in moment redistribution at ultimate with varying *A_f_*/*A_r_*. A list of elastic, ultimate moments and degree of redistribution in beams with different hybrid levels under symmetrical/unsymmetrical loading is presented in Table 1. It is seen that in the case of symmetrical loading, moment redistribution decreases as *A_f_*/*A_r_* increases. In the RC beam with steel bars (*A_f_*/*A_r_* = 0.0), the degree of redistribution is 26% at center support and −15% at midspan. Increasing *A_f_*/*A_r_* from 0.0 to 0.25, 0.5, 0.75 and 1.0 leads to a decrease in moment redistribution by 34%, 47%, 56% and 62%, respectively. In the case of unsymmetrical loading, the effect of *A_f_*/*A_r_* on moment redistribution is ignorable. The degrees of redistribution are around 9% at center support and −3.5% at midspan, irrespective of the *A_f_*/*A_r_* ratio. At *A_f_*/*A_r_* = 0.0 (i.e., RC beams with steel bars), unsymmetrical loading leads to significantly lower moment redistribution than symmetrical loading. The redistribution difference between symmetrical and unsymmetrical loading is substantially reduced as *A_f_*/*A_r_* increases.

## 4. Evaluation of Simplified Equations for Predicting Moment Redistribution

**Figure 16:**
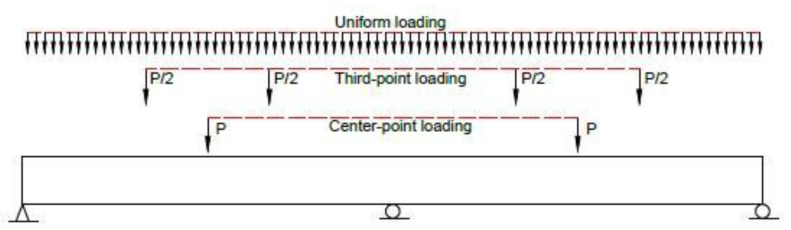
Beams under different types of loading.

In this section, available simplified equations based on the strain in tensile bars, *ε_t_*, for predicting moment redistribution at the support section are evaluated. As the support section is non-critical when unsymmetrical loading is applied, only symmetrical loading is considered herein. Apart from center-point loading as used in the previous section, uniform loading and third-point loading are also considered in this part of investigation, as illustrated schematically in Figure 16.

The ACI code [35] recommends the following equation to calculate the allowable moment redistribution:

(5) $$\beta_{u}=(1000\varepsilon_{t})\%$$

with a maximum of 20%, and *ε_t_* should not be smaller than 0.0075.

**Figure 17:**
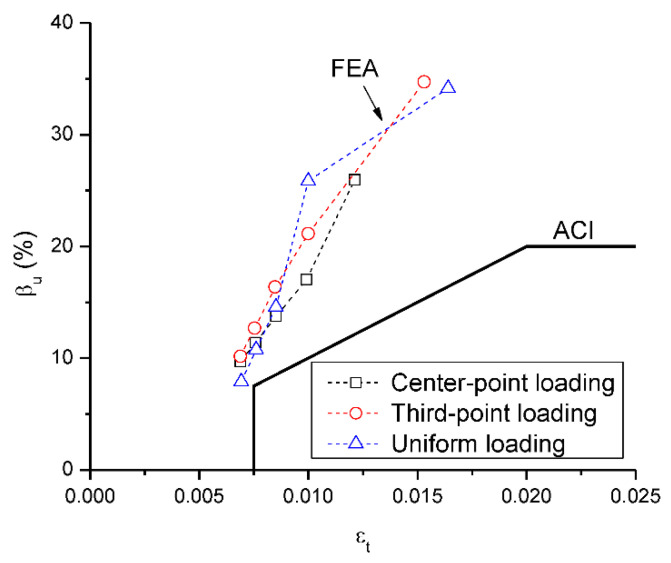
Relationship between *β_u_* and *ε_t_* according to FEA and ACI.

Figure 17 shows the FEA data regarding the *β_u_*-*ε_t_* relationship at center support along with the ACI curve. According to FEA, the β_u_ value increases with increasing *ε_t_* and the *β_u_*-*ε_t_* relationships by different types of loading exhibit good consistency. As far as the β_u_-*ε_t_* relationship is concerned, the ACI code is coincident to the FEA data, showing that this code can reflect the tendency of the redistribution variation with the *ε_t_* level. In addition, all of the FEA data are located above the ACI curve, suggesting that the ACI code is conservative. However, the code seems to be over-conservative at a high *ε_t_* level, partly attributed to the neglect of the stiffness difference between the critical sections.

Lou et al. [36] modified the ACI equation by introducing the parameter *ρ_r_*_2_/*ρ_r_*_1_ for predicting moment redistribution in continuous RC beams:

(6) $$\beta_{u}=(1000\varepsilon_{t}\lambda )\%$$

(7) $$\lambda =0.68-4.21\ln(\rho_{r2}/\rho_{r1})-2.05\ln^{2}(\rho_{r2}/\rho_{r1})$$

**Figure 18:**
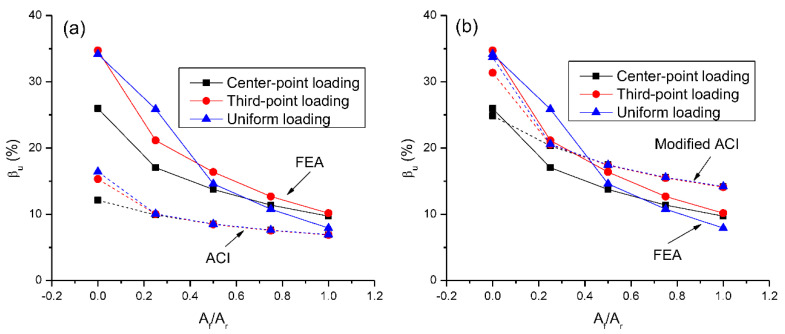
Comparison of *β_u_*-*A_f_*/*A_r_* relationships by FEA and simplified equations: (**a**) ACI equation; (**b**) modified ACI equation.

Figure 18a,b show a comparison of the FEA-predicted *β_u_*-*A_f_*/*A_r_* relationship with predictions by the ACI and modified ACI equations, respectively. The values of *M_u_*, *M_e_*, *ε_t_* and *β_u_* are presented in Table 2. It is seen that both the ACI and modified ACI equations can reflect the influence of *A_f_*/*A_r_* on moment redistribution in RC beams. In general, these equations can also reflect the influence of load type on moment redistribution, except at *A_f_*/*A_r_* = 0.25. According to FEA, in the case for *A_f_*/*A_r_* = 0.25, center-point loading leads to substantially lower moment redistribution than uniform loading. This is inconsistent with the predictions by simplified equations, which show that the redistribution values by different types of loading are almost identical. In addition, it is observed in Figure 18 and Table 2 that the ACI code underestimates the moment redistribution in continuous beams, especially at a low *A_f_*/*A_r_* ratio. The modified ACI equation shows an accurate prediction although it slightly overestimates the moment redistribution at a high *A_f_*/*A_r_* ratio.

**Figure 19:**
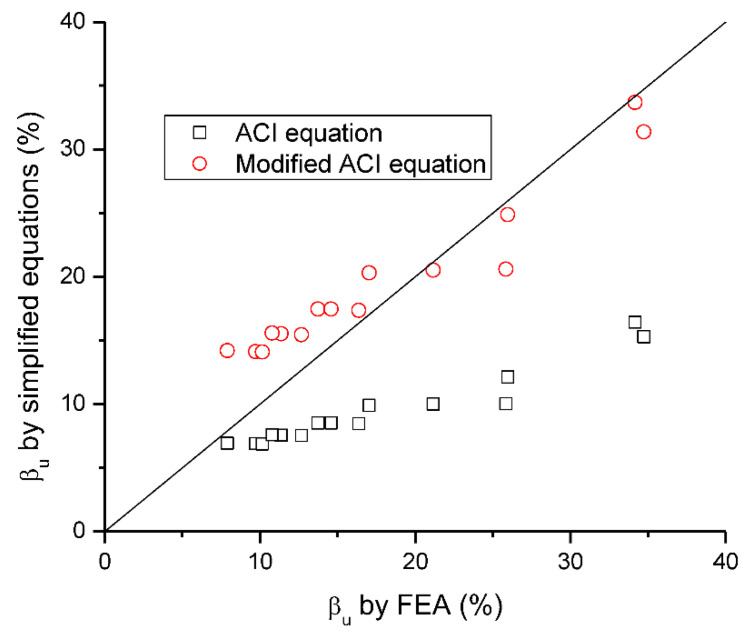
Correlation of *β_u_* by simplified equations with FEA.

Figure 19 shows a correlation of FEA-predicted values of *β_u_* with the ACI and modified ACI equations. It is observed that the ACI equation shows a poor correlation with the FEA data although its predictions are safe. The modified ACI equation exhibits much better correlation than the ACI equation but some of the data are in the unsafe side.

## 5. Conclusions

A numerical work was carried out to evaluate the flexural behavior of two-span RC beams with CFRP-steel hybrid bars. The main investigated variables were the hybrid level (*A_f_*/*A_r_* = 0.0–1.0), the load pattern (symmetrical and unsymmetrical loading) and the load type (center-point, third-point and uniform loading). The following conclusions can be drawn:

- Using hybrid or CFRP bars can effectively mitigate the cracking concentration of beams. Increasing *A_f_*/*A_r_* leads to a reduction in crack width in the critical section at ultimate.
- Increasing *A_f_*/*A_r_* leads to a reduction in yielding load and moment. The ultimate load is enhanced by 16.0% at symmetrical loading and by 11.3% at unsymmetrical loading, when *A_f_*/*A_r_* increases from 0.0 to 0.25. With continuing increases in *A_f_*/*A_r_*, the enhancement of the ultimate load is rather limited.
- At symmetrical and unsymmetrical loading, beams with *A_f_*/*A_r_* of 0.25 show 11.7% and 16.3% higher ultimate deflection than beams with *A_f_*/*A_r_* of 0.0, respectively. As *A_f_*/*A_r_* increases from 0.25 to 1.0, the change in ultimate deflection appears to be unimportant. Unsymmetrical loading leads to 6.0–15.0% higher ultimate deflection than symmetrical loading.
- A higher *A_f_*/*A_r_* ratio leads to significantly lower moment redistribution at symmetrical loading. The redistribution value is decreased by 62% as *A_f_*/*A_r_* increases from 0.0 to 1.0. By contrast, the influence of *A_f_*/*A_r_* on moment redistribution appears to be marginal at unsymmetrical loading.
- The influence of *A_f_*/*A_r_* and load type on moment redistribution is generally reflected in the ACI and modified ACI equations. The ACI equation shows conservative prediction on moment redistribution but appears to be over-conservative at a low *A_f_*/*A_r_*. The modified ACI equation is much more accurate than the ACI equation for predicting moment redistribution in those beams.

## Figures and Tables

**Table 1:** Values of *M_e_*, *M_u_* and *β_u_* in beams with different hybrid levels under symmetrical/unsymmetrical loading.

Load Pattern	*A_f_*/*A_r_*	*M_e_* (kN·m)		*M_u_* (kN·m)		*β_u_* (%)	
		Midspan	Support	Midspan	Support	Midspan	Support
Sym.	0.0	749.6	−868.8	862.4	−643.3	−15.04	25.96
	0.25	867.3	−1006.1	953.0	−834.7	−9.88	17.04
	0.5	920.5	−1069.7	993.9	−922.8	−7.98	13.73
	0.75	936.3	−1090.5	998.3	−966.5	−6.62	11.37
	1.0	935.3	−1092.0	988.3	−985.9	−5.67	9.71
Unsym.	0.0	838.8	−643.7	863.4	−594.6	−2.93	7.63
	0.25	931.9	−715.2	964.5	−649.9	−3.50	9.13
	0.5	955.0	−734.1	989.2	−665.8	−3.57	9.30
	0.75	968.9	−746.1	1003.1	−677.8	−3.53	9.16
	1.0	973.9	−751.5	1008.2	−682.8	−3.52	9.13

**Table 2:** Comparison of results in relation to moment redistribution in beams with different hybrid levels and load types.

Load Type	*A_f_*/*A_r_*	*M_u_* (kN·m)	*M_e_* (kN·m)	*ε_t_* (%)	*β_u_* (%)		
					FEA	ACI	Modified ACI
Center-point	0.0	−643.3	−868.8	1.21	25.96	12.13	24.86
	0.25	−834.7	−1006.1	0.99	17.04	9.91	20.31
	0.5	−922.8	−1069.7	0.85	13.73	8.52	17.47
	0.75	−966.5	−1090.5	0.76	11.37	7.58	15.53
	1.0	−985.9	−1092.0	0.69	9.71	6.89	14.13
Third-point	0.0	−673.5	−1031.8	1.53	34.72	15.30	31.36
	0.25	−855.0	−1084.4	1.00	21.15	10.02	20.53
	0.5	−934.4	−1117.2	0.85	16.37	8.47	17.37
	0.75	−975.8	−1117.4	0.75	12.67	7.54	15.45
	1.0	−996.1	−1108.7	0.69	10.15	6.88	14.10
Uniform	0.0	−702.5	−1067.2	1.64	34.17	16.43	33.68
	0.25	−881.7	−1189.2	1.00	25.86	10.05	20.59
	0.5	−962.7	−1126.9	0.85	14.58	8.52	17.46
	0.75	−1007.0	−1128.6	0.76	10.78	7.60	15.58
	1.0	−1025.9	−1114.0	0.69	7.91	6.92	14.19

## Data Availability

Not applicable.
